# Active Mapping and Robot Exploration: A Survey

**DOI:** 10.3390/s21072445

**Published:** 2021-04-02

**Authors:** Iker Lluvia, Elena Lazkano, Ander Ansuategi

**Affiliations:** 1 Autonomous and Intelligent Systems Unit, Fundación Tekniker, 20600 Eibar, Gipuzkoa, Spain; ander.ansuategi@tekniker.es; 2 Robotics and Autonomous Systems Group (RSAIT), Computer Science and Artificial Intelligence Department, Faculty of Informatics, University of the Basque Country (UPV/EHU), 20018 Donostia, Gipuzkoa, Spain; e.lazkano@ehu.eus

**Keywords:** mobile robots, mapping, exploration, frontiers, next best view, path planning

## Abstract

Simultaneous localization and mapping responds to the problem of building a map of the environment without any prior information and based on the data obtained from one or more sensors. In most situations, the robot is driven by a human operator, but some systems are capable of navigating autonomously while mapping, which is called native simultaneous localization and mapping. This strategy focuses on actively calculating the trajectories to explore the environment while building a map with a minimum error. In this paper, a comprehensive review of the research work developed in this field is provided, targeting the most relevant contributions in indoor mobile robotics.

## 1. Introduction

The problem of mobile robot navigation has been historically faced by decomposing it in three sub-problems: environment mapping, localization and trajectory planning. Those three sub-problems have developed into broad research areas over decades, tackled separately and in a deterministic way, ignoring the uncertainty in robot sensing and motion. In the early 1990s, probabilistic robotics turned the classical approach to navigation upside down, by developing algorithms that were able to explicitly take into account the intrinsic uncertainty in robot sensors [1,2]. Probabilistic robotics focuses on representing the uncertainty and information by probability distributions and not on the basis of a single guess. With the rise of those methods, the problem of Simultaneous Localization And Mapping (SLAM), also known as Concurrent Mapping and Localization (CML) [3,4] arose: the mapping of sensor readings with respect to a global frame of reference depends on the robot’s location in that frame and on the uncertainty in this robot’s pose due to the cumulative odometry error that affects the process of building the map. Visual odometry allows for enhanced navigational accuracy in robots or vehicles using any type of locomotion. Combining visual information from cameras or inertial sensors with wheel motion information allows us to overcome the drift and provides much more accurate localization [5]. However, errors in the built map and robot’s pose are correlated. Thus, these two problems should be tackled together. SLAM techniques vary depending on whether indoor or outdoor robots are used. Outdoor environments are more challenging as they do not have specific limits and the criteria of choosing the next navigation point or termination can be completely different. Besides, indoor and outdoor classic SLAM systems differ from each other in aspects such as the individual requirements, sensors provided and morphology of the robots, Markovian, Kalman filter-based and particle filter-based are the most common techniques used to solve the SLAM problem. Although SLAM in static environments can be considered as solved [6], dynamic environments are still challenging.

SLAM can be considered to be a mapping process in which robot localization is uncertain. However, the planning task is put aside during the map building process. Although strategies like coastal navigation can be used [7], while building the map the robot is usually guided by a human by means of teleoperation. In this way, the wheels’ drift can be minimized, ensuring as well the full coverage of the environment. Once the map is available, stochastic planning techniques, e.g., Partially Observable Markov Decision Process (POMDPs), are used for navigation.

The advent of SLAM techniques motivated huge advances and opened new possibilities for robot development, but there are still considerable challenges in performance when adding environment dynamism or increasing dimensionality. Moreover, teleoperating the robot for mapping is usually a highly time-consuming task, especially in large areas or when the movement of the robot is limited. In other cases, it is difficult or even impossible for the robot to be guided due to insufficient connectivity or dangerous conditions, such as in rescue operations of natural disasters.

Active SLAM, hereinafter called ASLAM, is the task of actively planning robot paths while simultaneously building a map and localizing within it [8]. ASLAM goes a step further than the classic SLAM problem as it seeks the robot to move autonomously during the whole mapping process. ASLAM could simplify the setting up of a navigation system in many applications, as the robot would be capable of building the map by itself with no human interaction.

To the authors’ best knowledge, the literature lacks a survey in ASLAM, with the exception of very specific subtopics in some publications, such as [6]. This is the main motivation for the present work. We expect to provide an overall perspective of this complex problem and at the same time guides active researchers in the path towards the desired solution. We will focus on studies done in static indoor environments and using a single robot. Although this task can be completed using several robots, articles oriented to multi-robot exploration fall out of the scope of this review. Pursuing the clarity and practicality for the reader at the time of giving information about ASLAM, this article is organized as follows: Section 2 briefs the historic development of the problem; Section 3 details the three iterative steps any ASLAM strategy consists of; Section 4 details the optimization strategies present in the literature in order to improve the performance of already existing solutions. It follows Section 5, in which place revisiting actions proposed by the community are described. Differences related to world dimensionality are described in Section 6, together with problems related to computation. Section 7 summarizes the differences among the techniques expounded in this work. Section 8 outlines the ongoing developments together with the open research questions. Finally, Section 9 concludes the paper providing alternatives for further research.

## 2. Historical Overview

It was not until 20 years after the first mobile robots emerged in the decade of 1940 [9], that Shakey, a general-purpose mobile robot, capable of reasoning about its own actions, was built [10]. Since then, many mobile robots have been developed for a wide variety of applications [11,12,13,14].

However, robots need to navigate in order to perform tasks autonomously. Mobile robot navigation is not yet a solved problem, although a large number of algorithms have been developed for mapping, localization and trajectory planning. As mentioned in the introduction, mapping and localization were initially studied separately. Later on, they were identified as dependent on one another and the problem began to be known as SLAM. Then, SLAM-based systems widely proliferated to other areas such as vision-based online 3D reconstruction or self-driving cars. As each area has its respective requirements, the sensors used as source of information may differ too, although most of them can be categorized in range sensors and vision-based systems, either in a 2D or a 3D system. Many other sensors can be configured as the main sources of information or act as additional ones, e.g., Global Positioning Systems (GPS), rotatory encoders, sonars, or Inertial Measurement Units (IMU) [3,15,16,17]. Nevertheless, many researchers opt for lasers or cameras because a big amount of robots are intended to operate in indoor GPS-denied environments, and other sensors are not usually accurate enough. IMUs are incorporated in a multitude of vehicles as additional sensors as, comparing to other devices, they offer relevant information about rotational axes. In fact, some cameras integrate IMUs inside themselves already (https://www.intelrealsense.com/depth-camera-d435i/, accessed on 11 February 2021).

It is worth recalling that in standard SLAM processes the robot is normally guided by a human in order to ensure that the environment is fully covered and that the loop is closed. In fact, the problem of recognizing an already mapped area typically after a long exploration phase is known as loop detection and it is one of the biggest problems in SLAM processes [18]. Loop closure refers to exploiting the detection of an already mapped area to minimize the accumulated error [19] during the navigation. It is also the responsibility of the operator to decide the trajectories of the robot and the termination criteria, factors that directly affect the quality of the resulting map and, in consequence, in the performance of the corresponding navigation. Consequent local drifts are accumulated during motion, increasing the uncertainty and leading to an inaccurate estimate. Loop closure enhances considerably the veracity of the map with respect to the traversed path, as the technique attempts to correct this divergence in the position of the robot. Identifying previously visited locations can also be relevant when addressing the global localization problem, and it can ease the recovery of a kidnapped robot. Loop closure detection methods differ from one another as they may be geared towards specific map representations [20,21,22,23,24,25]. Despite all this, the resulting model usually requires a refining process to correct any erroneously added element. This is normally a consequence of the sensor noise and the drift of the mobile robot. This post-processing can be done manually or automatically [26,27,28].

Exploration while mapping or ASLAM pretends to overcome the disadvantages and potential sources of incongruity previously mentioned by developing methods to perform the exploration step automatically, i.e., automatizing the robot guiding process by selecting online the paths or navigation sub-goals that will lead to a more accurate map. The exploration field, extensively studied in the last decade [29,30,31,32,33], offers great advantages for mobile robots, especially in hostile environments.

It has also been referred to in scientific literature as automatic SLAM [34,35], autonomous SLAM [36,37,38], adaptive CML [4], SPLAM (Simultaneous Planning, Localization and Mapping) [39] or robot exploration [40].

### ASLAM in Different Research Fields

This concept of “active motion selection” is addressed from two research areas: mobile robotics and computer vision. In robotics [41], exploration refers to the autonomous creation of an operational map of an unknown environment. Besides generating a world representation while dealing with uncertain localization, the robot must control its motion. Namely, it must calculate the goals and the actions to take thereon to achieve those goals while actively reacting to unexpected situations.

In computer vision, the problem emerged as active perception [42] and it is defined as an intelligent and reactive process for gathering information from the environment to reduce the uncertainty around an element. It is considered intelligent because the sensor’s state changes on purpose according to the sensing strategies. Active perception applied to mobile robotics describes the capability of the robot to determine particular movements in order to get a better understanding of the environment. In the context of localization, the position and/or heading of the sensor is moved in an attempt to search for signs that reduce the position uncertainty. In consequence, the shape, geometries or appearance of the surrounding elements cannot be unknown and the robot must be able to recognize them with accuracy. This is called active localization and, although the environment is assumed to be already mapped, many advances in this field can be extrapolated to the ASLAM problem studied in this review [43,44,45]. A work that is especially worth highlighting is the one presented by Zhang et al. [46]. On the one hand, they present a perception-aware receding horizon planner for micro aerial vehicles that allows the robot to reach a given destination and avoid visually degraded areas at the same time [47]. On the other hand, they define a dedicated map representation for perception-aware planning that is at least one order of magnitude faster than the standard practice of using point clouds [48,49]. Both contributions could push the research of mapping unknown areas in the right direction. In contrast, active perception in the context of mapping refers to the challenge of modeling the environment with no prior information and calculating the motion required to achieve it. Thus, this idea includes two processes, the reconstruction of the environment itself and the motion control of the sensor. This method is referred to as active mapping, i.e., a map of the unknown environment must be built in a finite time, optimizing the accuracy of the resulting model and the actions executed for that purpose. There are several methods for estimating the spatial transformation that corresponds to a specific motion. Approaches based on probabilistic filters [1,50] give good results and are thus, used most commonly together with those that employ Structure from Motion (SfM) [17,51]. SfM is a photogrammetric range imaging technique for estimating 3D structures from 2D image sequences, similar to estimating the structure from stereo vision.

Some of the problems ASLAM is facing have already been tackled by other research fields. For instance, active vision deals with the task of choosing the next optimal sensor pose for 3D object reconstruction or obtaining a complete model of a scene. This is known as Next Best View (NBV) and it has been studied since the 1980’s [52]. Alternatively, computational geometry confronts the art gallery problem. This problem was first set out by Victor Klee in 1973 [53] as a matter of interest in the field of security. The art gallery problem for an area *A* is to find a minimum-cardinality covering viewpoint set *P* for *A*. It was called this way because one envisions the area *A* as the floor plan of an art gallery, and the points in *P* as locations to place guards, so that every part of the art gallery is seen by at least one guard. In summary, provided that the system already has a digital model of the environment, the objective is to find the minimum set of poses from which all the scene is seen. Transferred to computational geometry, the gallery corresponds to a simple polygon and each guard is represented by a point in the polygon. The goal is then to find a minimal cardinality set of points (guards) that can be connected by line segments without leaving the polygon. Many contributions have been done finding the optimal placement of surveillance devices [54,55,56,57,58].

Whatever the context, the problem consists of fully covering a partially-unknown environment. In the next section, ASLAM is explained in detail and its differences with respect to other similar concepts are discussed.

## 3. The ASLAM Problem

**Figure 1:**
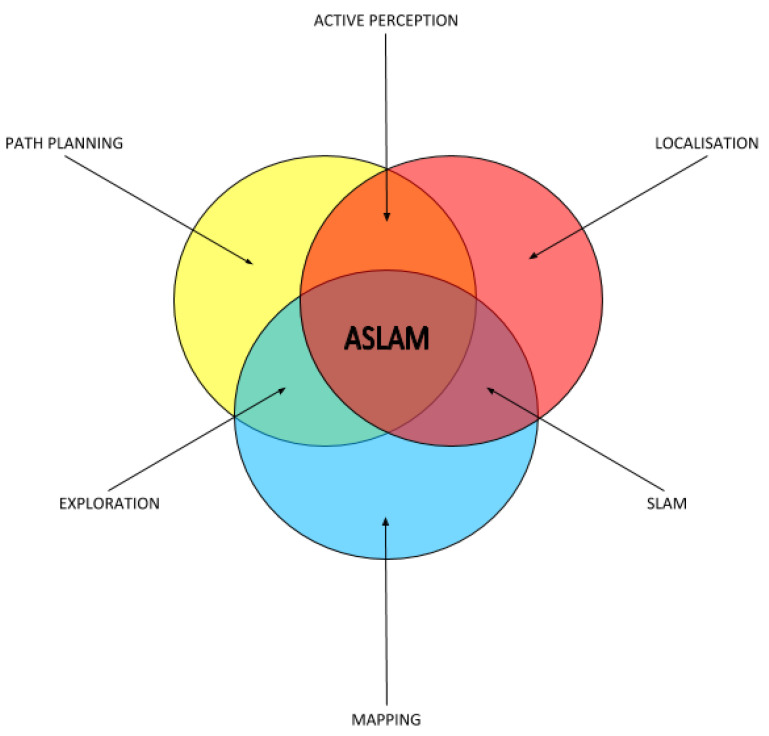
Components that form the Active Simultaneous Localization And Mapping (ASLAM) problem, expressed as set theory. Overlapping areas represent the combination of individual tasks.

According to the literature, an ASLAM algorithm consists of three iterative phases [59]: pose identification, goal selection, and navigation and checking. These phases involve the classic tasks of localization, path planning and mapping, whose relationship is shown in Figure 1. The pose identification step identifies the possible destinations; the optimal goal selection phase selects the optimal destination; and, in the last stage, remaining candidate points affect the algorithm termination. As it can be seen, the three phases depend on the candidate viewpoints. Those candidates are just possible destinations of the mobile robot, which are limited by the model used to manage the navigation component. Therefore, how the environment data is represented may have a huge impact. Appendix A summarizes environment representation alternatives. Readers non-familiar with the robot mapping process are referred to it to better understand the techniques described further on in this paper.

### 3.1. Pose Identification

Given the partial map of the environment, the robot identifies a set of destinations. In theory, all the physically reachable destinations should be evaluated, as they are potential gains of information and, thus, any of them could be the desired optimal viewpoint. However, in practice, the computational complexity of the evaluation grows exponentially with the search space which proves to be computationally intractable in real applications [39,60,61]. Thus, the implementation of certain filters reduce the number of candidates and the complexity of the problem [62,63]. Some poses can be discarded a priory, such as the ones that are already tested, the ones surrounded by already mapped points or the ones that are too far in the unknown space.

The most straightforward approach could be one that sets random destinations to an existing SLAM system until a certain condition is met. In this case, the ASLAM solution would not differ much from a SLAM technique. However, researchers have developed more elaborated algorithms to autonomously map an environment.

**Figure 2:**
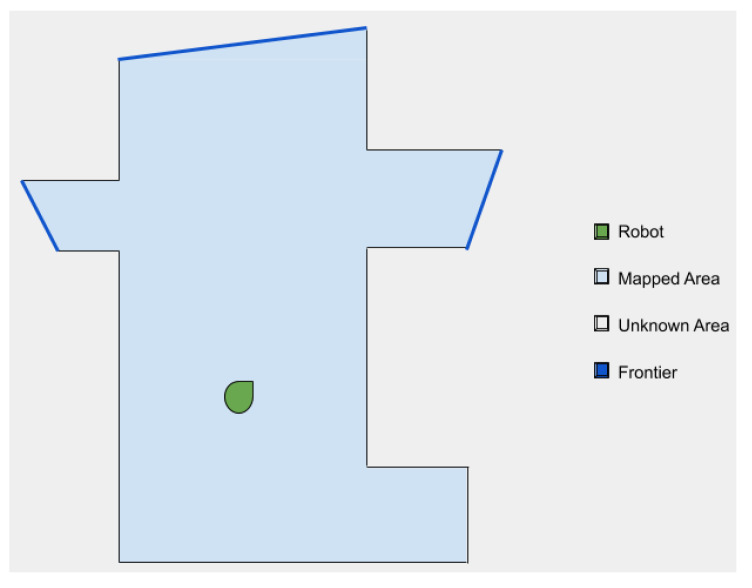
Example of a 2D map with frontiers.

Concerning dimensionality reduction and mapped space, a concept that is widely used in the context of exploration is that of a frontier [40,64,65]. Frontiers are regions on the boundary between open space and unexplored space, i.e., points in the map between the free known space and the unknown space. The main idea behind these points is that, as they are in the mapped space, it is very probable that the robot can reach them. In addition, they ensure that unexplored areas next to them can be covered. Frontiers, thus, represent optimal sets of points to be reached in order to expand the explored environment. An example of a map with all the frontiers identified is shown in Figure 2.

Frontiers can be searched by applying computer vision techniques such as edge detection or region extraction [66]. However, the vast majority of the approaches find these points with algorithms based on Breadth-First Search (BFS) [67,68,69] and cluster these frontiers to be more efficient, as adjacent frontier points can lead to a similar exploration result proportional to the granularity of the map. Generally, the clustering is performed using k-means [70,71,72,73], although some authors propose other alternatives such as histogram-based methods [74].

### 3.2. Optimal Goal Selection

Once the set of potential destinations is identified, the cost and gain of each of them need to be estimated. The cost represents the effort required to reach the actual goal, e.g., the distance between the actual position and the target pose. The gain corresponds to the difference between the information provided by the map after and before navigating to the selected goal. Information usually refers to the number of points discovered. Generally, gain and cost are variables of a function that results in a value called utility [75]. Utility serves as a metric to compare exploration trajectories [76,77]. Ideally, to compute the utility of a given action, the robot should reason about the evolution of the posterior over the robot pose and the map, taking into account future (controllable) actions and future (unknown) measurements. However, computing this joint probability analytically is, in general, computationally intractable [78,79,80], and thus, it is approximated [78,81].

### 3.3. Navigation and Checking

The robot navigates to the chosen optimal destination and updates the map during motion or upon attaining the goal. Updating a map means completing unknown data as well as improving the quality of the already known elements or correcting old or erroneous information. Originally, the navigation is performed with a classic technique, such us Dijkstra or A* [82], which is completely independent of robot exploration. However, some researchers propose adapting these trajectories for the autonomous mapping purpose, as it is described in Section 4.2 and Section 5. Then, the robot checks if the exploration procedure must continue. In that case, the process proceeds again with the pose identification step and another iteration is executed.

When exploring an unknown environment, there may always be potential areas of mapping or accuracy improvement, driving the system to an infinite loop. In order to perform the autonomous mapping in a finite period of time, a termination criteria is defined. Thus, the procedure will be considered finished when that criteria is satisfied [39,83]. Many exploration systems based on frontiers consider the exploration process as concluded when no frontier targets exist in the map [78,84,85]. Other works could opt for more straight-forward solutions as the distance traveled, the time elapsed or the number of frames captured. Yet these conditions are independent of the quality or completeness of the model built, and their effectiveness is highly dependent on the optimal candidate selector. Kriegel et al. [86] propose a 3D reconstruction system in which the termination criteria is a predefined maximum number of scans mapped. This value is set based on an estimation of the quality and the completeness rate of the scene, and it is unique for each area due to the particular properties of different objects. Hence, there are stopping conditions that are environment-specific.

Uncertainty metrics from Theory of Optimal Experimental Design (TOED) [87] seem promising as stopping criteria, compared to information-theoretic metrics which are difficult to compare across systems. However, this decision is currently an open challenge [6].

## 4. Optimization Trends

Improving the phases described in Section 3 (pose identification, optimal goal selection and navigation and checking) can reduce the time needed to finish the procedure, shorten the distance traveled, or enhance the fidelity of the resulting map [88,89,90]. Thus, different techniques have been developed, which may focus on one stage or another. One relevant point among active mapping approaches is whether it attempts to calculate the optimal exploration viewpoint [91] or, in addition, it calculates the optimal trajectory to reach that viewpoint [78].

Besides, all the currently available map is processed each time to find the possible destinations. This process requires an increasing amount of time due to the enlargement of the available map. To cope with this limitation, the optimal pose can be calculated while the map gets updated, and a new destination can be set before reaching the actual one [92]. Or those decisions can be delayed until the robot has achieved the navigation goal and it is waiting for the next one [93]. Besides, most algorithms focus on the coverage of the area at the time of motion planning [94], but some of them take into account the quality of the mapped sections too [95].

Most of the frontier-based exploration approaches, for example, optimize only the navigation goal. They find frontiers, which are usually clustered, and navigate to them. While the focus is set in the frontier extraction, clustering or prioritization strategy, the navigation techniques generally are completely independent of robot exploration. Sampling-based ASLAM methods arise as an alternative to frontier-based exploration algorithms. These techniques randomly generate robot states and calculate the path which maximizes the information gathered during the navigation [96].

Moreover, many strategies of recent years not only attempt to find the optimal pose for exploration, but also the optimal trajectory that maps the environment more efficiently. These two aspects are discussed deeply later in this section.

### 4.1. Approaches for Pose Optimization

The first exploration attempt can be attributed to Yamauchi [64]. He proposed an approach based on frontiers in a grid cell map. As explained in Section 3.1, any free cell adjacent to an unknown cell is considered a frontier edge cell. These cells are grouped into frontier regions and the robot attempts to navigate to the nearest accessible, unvisited frontier. The path planner uses a depth-first search on the grid map to calculate the shortest obstacle-free path from the robot’s current cell to the cell containing the goal location. By moving to new frontiers, a mobile robot can extend its map into new territory until the entire environment has been explored. This strategy has been of great success and many researchers have developed frontier-based exploration methods.

Dornhege et al. [97] extend Yamauchi’s 2D frontier-based exploration method towards 3D environments by introducing the concept of voids, which are unknown 3D volumes. They focus on solving the problem of selecting NBV configurations for a 3D sensor carried by a mobile robot platform that is searching for objects in unknown 3D space but the robot platform stands still. On the one hand, frontier cells are clustered by a union-find algorithm forming the frontier cluster set and, on the other hand, the set of void cells contains all unknown cells that are located within the convex hull of the accumulated point cloud represented by an octomap. Locations that are not reachable by the sensor are removed using capability maps, which are representations that include information about the possible movements that the robot can accomplish [98]. The reachable frontier cells are directly sorted by the volume of the void space that would be discovered and then the NBV planner identifies the configuration of the sensor from which the maximal amount of void space can be observed. The computation stops after a determined number of valid poses have been computed.

Zhu et al. [99] extend the traditional frontier-based exploration to 3D and implement their approach in the Robot Operating System (ROS) framework [100] on board of a Micro Aerial Vehicle (MAV). The 3D map of the environment being explored is built incrementally from two consecutive point clouds, represented by means of octrees with free, unknown and occupied cells (see Appendix A). They group the continuous cells into several clusters and select their geometric central cell as the representative for this candidate frontier. In the presence of multiple candidate frontier cells, the optimal goal frontier is determined by criteria that take into account the new information gain and the cost of moving to it. Their implementation is available publicly (https://github.com/zcdoyle/fbet, accessed on 11 February 2021).

While most of the exploration-exploitation works are based on free-space frontiers, Senarthane and Wang [101] present a 3D environment exploration strategy based on the concept of surface frontiers. They define a surface frontier voxel in a 3D occupancy grid map as a boundary voxel of a mapped surface where at least one of the six faces of the voxel is a frontier, i.e., is exposed to the unmapped space. Hence a boundary voxel is a traditional frontier extrapolated to the geometric boundary of a mapped surface. Then, they follow the common procedure of finding the frontier representatives from the 3D occupancy grid map, generating the valid view configurations and selecting the optimal view based on utility criteria. It is shown that generating surface frontiers is computationally less expensive than generating free-space-based frontiers.

Usually, common approaches direct robots to frontier edges for exploration, that is, they actively search for areas between known and unknown spaces to set them as navigation goal candidates. Alternatively, some researchers present works where frontiers are found indirectly combining other techniques [63,84]. For instance, Dai et al. [84] present a hybrid exploration approach between frontier-based and sampling-based strategies. Authors identify the regions that exploration should focus on using frontiers and, then, it samples candidate views avoiding the clustering of the individual voxels into larger frontiers, thus reducing the associated computational cost.

Ravankar et al. [85] equip a UAV with an IMU for a 9 DoF position estimate, a barometer for altitude control, and a Microsoft Kinect that serves both as an RGBD camera and as a 2D LIDAR sensor. Then, an Extended Kalman Filter (EKF) is used to fuse all the sensor data into single navigation information to control the velocity, orientation and position along with sensor error bias. The key aspect of the work by Ravankar et al. is to test whether mapping and exploration can be performed by using low-cost RGBD sensors. That is, Yamauchi’s [64] frontier detection is used for exploration, GMapping [102,103] for mapping and localization, DWA [104] for navigation and a spatial alignment [105] to transform the 3D gathered data into a dense 3D map.

### 4.2. Approaches for Trajectory Optimization

Rapidly-exploring Random Trees (RRTs) [106] with a Receding Horizon (RH) strategy are well known sampling-based alternatives to frontiers. These strategies are so-called because the prediction horizon keeps being shifted forward, in the same way, the environment is discovered and the map updated. RRTs are commonly used for path planning in predictive control models [107,108,109], but they can also be successfully applied in ASLAM approaches. RRTs show a heavy tendency to grow towards unknown regions, making them ideal to passively detect frontiers for exploration. Because of this natural exploratory behavior, the leaves of the tree of an RRT planner are potential frontier points that must be analyzed. They can be considered as points of an efficient exploration trajectory itself.

The planner proposed by Bircher et al. [83] employs a Receding Horizon Next Best View (RH-NBV) scheme where an online computed random tree finds the best branch, the quality of which is determined by the amount of unmapped space that can be explored. The exploration is considered solved when the number of nodes of the tree is bigger than a tolerance value, while the gain of the node with the highest gain remains zero. In an extension of that work [110], Bircher et al. present another sampling-based receding horizon path planning paradigm that is not limited to volumetric exploration but also addresses the problem of autonomous inspection. The objective of the presented planner is to generate paths that cover an unknown volume or inspects a surface, depending on the objective function. The employed representation of the environment is again an octree with free, occupied and unknown space.

Umari and Mukhopadhyay [62] make use of RRTs to grow towards unknown regions and passively detect frontiers. Nevertheless, the tree is not used to define the robot trajectory itself, but to search for frontier points. It runs independently from robot movement. On this basis, the authors divide the exploration strategy into three modules: the RRT-based frontier detector module, the filter module, and the robot task allocation module. The first detects frontier points and passes them to the filter module. The filter module clusters these points and stores them, using the mean shift [111] clustering algorithm. In this step, the invalid and old frontier points are deleted too. The last module, i.e., the task allocation module, receives the clustered frontier points and assigns them to the robot for exploration. Besides, an additional novelty of this work is the use of multiple trees growing independently to accelerate the searching process.

Papachristos et al. [112] present an uncertainty-aware exploration and mapping planning strategy that employs a receding horizon, two-step, planning paradigm. The method computes an optimized sequence of viewpoints for exploration of unknown spaces and its first viewpoint is selected to be visited. However, opposite to the works cited above, the path to this new viewpoint is computed through a second planning layer that aims to optimize the probabilistic mapping behavior of the robot and minimize the root’s belief uncertainty.

In this vein, in a more recent work by Papachristos et al. [113] propose two algorithms focused on autonomous unknown area exploration, a “Receding Horizon Next-Best-View planner” (nbvplanner) and “Localization Uncertainty-aware Receding Horizon Exploration and Mapping planner” (rhemplanner). They use an RH-NBV planner (named nbvplanner) in an environment represented in an occupancy grid map divided into cubical volumes, that can be marked as free, occupied and unmapped. The cells are marked as unmapped if the direct Line of Sight (LoS) does not cross occupied spaces and compiles with the sensor model. As in many other cases, the volumetric data is stored using an octomap. Then, a geometric RRT is incrementally built from the robot space, whose nodes collect an information gain value based on the unmapped volume and the path cost, giving preference to shorter paths. Its edges are given by collision-free paths. To cope with the uncertainty in robot localization, they propose a “Localization Uncertainty-aware Receding Horizon Exploration and Mapping planner” (rhemplanner) that replicates the steps done in nbvplanner until a finite-steps path that maximizes the exploration gain is identified. Then, in a second phase, a new path to reach that viewpoint is computed. This alternative trajectory ensures that low localization uncertainty belief is maintained. The complete process is iteratively repeated. A visual-inertial odometry framework is used to increase the robustness and accuracy of the methods. It also enables the localization uncertainty-aware planning, as belief propagation takes place so that the sampled paths contain the expected values and covariance estimates of both the robot state and the landmarks corresponding to the latest tracked features. For every path segment, the expected IMU trajectories are derived and used for the robot belief prediction, which takes place by running the state propagation step in the EKF-fashion of the visual-inertial odometry. The algorithms are tested in real scenarios [114]. Besides, both the code and the experimental datasets are released to allow systematic scientific comparisons.

Due to the benefits of RH-NVB planning and the classic frontier exploration planning, Selin et al. [63] propose to combine both techniques. They use the frontier exploration method for global exploration and RH-NBV planning for local exploration, assuming that an agent that uses an RH-NBV planner efficiently explores the nearby surroundings but it typically has a very low score when the goal is far away. Thus, tending to terminate the exploration prematurely. When the RH-NBV planner explores everything in its nearby surroundings, it caches nodes with high potential information from previous RRTs and considers them as planning targets, leading to a frontier exploration behavior. Once again, the potential information gain function is proportional to the unmapped area explored.

Following a completely different strategy with respect to the approaches discussed in the previous lines, Faria et al. [115] propose combining frontier exploration with Lazy Theta* path planning algorithm. Theta* is a variant of A* that propagates information along grid edges without constraining the paths to grid edges [116,117]. In the same way, Lazy Theta* is a variant of Theta* which uses lazy evaluation to perform only one line-of-sight check per expanded vertex [118]. They use any-angle path planning [119] to decrease the computation of the processing and the number of line of sight checks. In addition, this algorithm has been applied successfully in competitions with autonomous multirotors [120]. Another relevant aspect of this work is that they abandon the regular grid mindset entirely to take full advantage of the spacial clustering with sparse grids.

No matter the technique, the core idea of most of the ASLAM approaches is to give the robot the capability of building a map autonomously in an unknown environment. As a result, the robot will be able to navigate to familiar positions. However, this task cannot be fulfilled without a map. Nevertheless, Deng et al. [121] go a step further and propose the idea of reaching a goal without the requirement of previously having a map. They focus on tracking failure avoidance during vision-based navigation and present a framework for planning and traversing a path by a mobile robot toward a goal and through an unknown environment.

## 5. Place Revisiting

The uncertainty in robot localization and the degraded correspondence of the map with the real environment induced by the noise of the sensory devices and the lack of reliable references is aggravated when continuously exploring unknown areas. In some way, either searching for frontier points explicitly or implicitly, most of the ASLAM approaches to guide the robot to regions that are not yet visited. Their decision function’s objective is just to minimize the number of accessible frontiers, i.e., points to which the robot could navigate and where, potentially, information of unknown areas can be gathered. Nonetheless, these exploration algorithms maximize coverage disregard the cumulative effect of the localization drift, which can lead to unrepresentative maps. For this reason, many works concur that the navigation policy of ASLAM systems should reduce uncertainty by balancing exploration actions and place revisiting actions [122,123]. The place revisiting topic is also known as loop closure and it is also inherited from SLAM.

In that vein, González-Baños and Latombe [124] present an algorithm that instead of using frontiers, builds the map connecting successive safe regions, which are the largest regions guaranteed to be free of obstacles. The same map is used for planning safe motions too. Safe regions are used for an estimation of overlap between future measurements and the current partially-built map and to check that this overlap satisfies the requirement of the alignment algorithm. Finally, those regions enable the NBV algorithm to select the position that is the most likely to explore a large area of the environment, based on the information gain estimation of each new position candidate. In principle, the mapping process finishes when the boundary of the union of all the local safe regions contains no free curve. In practice, the algorithm stops when the length of each remaining free curve is smaller than a specified threshold, as it is better suited to handle complex environments containing many small geometric features.

Stachniss et al. [125] present a 2D approach for active loop-closing that aims to cope with the imperfect control and sensing during continuous exploration. They combine a Rao–Blackwellized particle filter for localization and mapping with a frontier-based exploration technique extended by the ability to actively close loops. The key concept is to force the robot to traverse previously visited loops again to reduce the uncertainty in the pose estimation and obtain more accurate maps.

In the 2D ASLAM algorithm proposed by Carlone et al. [78], the particle-based SLAM posterior approximation is evaluated using the Kullback–Leibler divergence [126] to decide between exploration and place revisiting. The metric is used in the estimation of the expected information from a policy, which calculates the NBV. Candidate targets include frontier targets and trajectory targets, which allow the robot to revisit places when filter uncertainty gets high. Nevertheless, as in previously seen cases, the exploration is considered finished when no frontier exists on the map. The expected information from a policy is the difference between the expected map information after a specific motion command and the current map information, understanding as map information the number of visited cells, i.e., cells with occupancy probability not equal to 0.5. The expected information gain is the sum of the information gain of each pose in the trajectory, normalized by its distance.

Valencia et al. [127] introduced the improvement of the revisitation of known areas with respect to a classical exploration method in combination with a SLAM technique. They present an active exploration strategy integrated with Pose SLAM [128], a variant of SLAM in which only the robot trajectory is estimated and where landmarks are used to generate relative constraints between robot poses. In Pose SLAM, a probabilistic estimate of the robot pose history is maintained as an exact sparse graph [129]. Alternatively, the Active Pose SLAM method evaluates the utility of exploration and place revisiting sequences and chooses the one that minimizes the overall map and path entropies. The approach minimizes entropy instead of maximizing coverage as only those trajectories with an entropy measure below a threshold are chosen as safe exploratory routes. In addition, there are actions, i.e., navigation goals, to reduce uncertainty by closing loops. Note that when evaluating the information gain over the map, only a very coarse prior map estimate is computed.

### Dynamic Environments

While most works assume that the environment is static, Trivun et al. [82] developed a 2D ASLAM system to map a dynamic indoor environment. The algorithm uses FastSLAM [130], a Rao–Blackwellized particle filter, for localization and mapping, whereas the A* search algorithm [131] is used in conjunction with the dynamic window approach (DWA) [104] for navigation. During the exploration, the robot first scans the global occupancy matrix for gaps and it detects areas that are orthogonal within several degrees to the robot’s position and within the range sensor’s scope. The areas that yield outside this group are marked as hidden areas to be always taken into consideration before edge frontiers, as they are closer and usually cheaper to explore. When there are several hidden areas, the algorithm picks the closest one. A hidden area can be visited twice if the system is incapable of completely mapping it. The first time, the algorithm sets that area as the last in the order of preference and marks it as visited. If it is visited once again, but still not mapped completely, it updates the costmap, so that the algorithm does not detect that gap again. If there are no hidden areas, edge frontiers are unique candidates for exploration. The goal is to make the robot orientation vector orthogonal to the edge, so that the sensor covers as much area as possible. Finally, the sum of the uncertainty of each candidate is calculated and the edge frontier gap with the highest value is selected. The program finishes when there are no gaps in the global costmap.

Mammolo [132] also tackles the problem of dynamic environments. More specifically, the work presents an ASLAM algorithm for static environments, with an extension for crowded environments. Although it does not introduce a novel technique for exploration or path planning itself, as an implementation of the classical frontier-based approach proposed by Yamauchi [64] is used for exploration, and the utility function is based on the Shannon and Rényi entropy developed by Carrillo et al. [133]. Mammolo assumes a static environment where the only dynamic objects are humans, and they implement an algorithm that filters moving people in the 2D range data so the ASLAM system can complete the task with static, reliable data. The experiments in crowded environments are not exhaustive enough but they do show that the integration of that filter into ASLAM systems can improve the performance.

## 6. Data Dimensionality and Computational Cost

**Figure 3:**
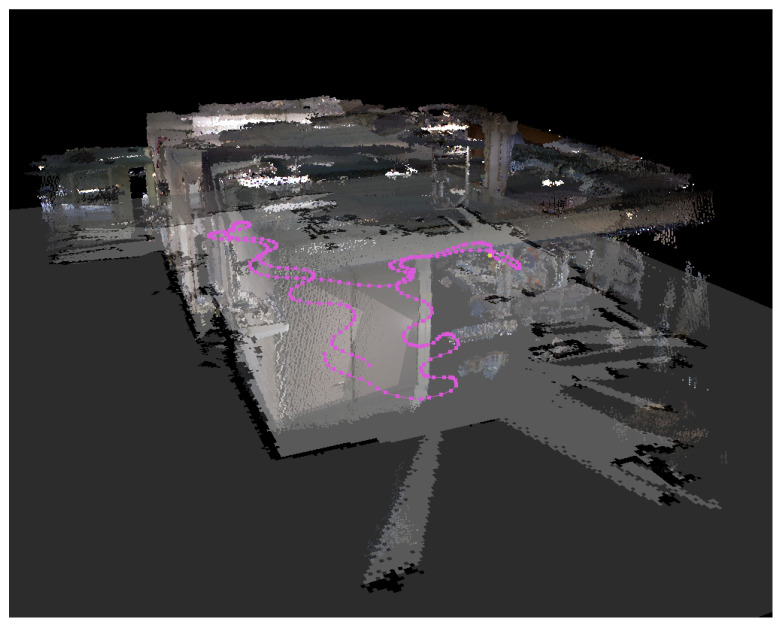
The 3D model of the robotics laboratory in Tekniker research centre and its corresponding 2D occupancy map. Pink points represent the trajectory followed to create them.

Although most of the 2D exploration algorithms were developed in the early 2000s, with the advent of 3D sensors (RGB-D vision systems, 3D range sensors, and so on), the possibility of 3D mapping arose. 3D environment reconstruction offers much interesting context information and extends the applications of robots, such as autonomous navigation of aerial robots, where 3D information is vital. Figure 3 shows an example of the 3D mapping of a robotic laboratory.

A decade later 3D ASLAM began to be viable and, thus, investigated. However, the huge computational cost requires a high-performance machine and efficient memory management [78,134,135] to make possible the execution in an acceptable period of time.

2D systems can meet the requirements in many applications, but 3D systems add definitely much valuable information and give robots new capabilities: 3D realistic models can be built, which may be used in simulation; volumetric information of objects is required for, e.g., mobile manipulation or collaborative tasks; detecting obstacles at different heights makes possible the management of difficulties such us slopes, steps and ditches, i.e., negative obstacles, enabling more flexible and secure navigation. It is true that these enhancements carry more complexity, but since the boom of 2D ASLAM more efficient algorithms have been developed and the hardware components’ computational power has considerably increased. In addition, 3D data management is a key requisite for Unmanned Aerial Vehicles (UAVs). UAVs are nowadays a valuable source of data for inspection, surveillance, mapping, and 3D modeling issues [136]. They are lightweight and cheaper compared with how much a ground navigation platform costs. Besides, the objectives of 3D ASLAM make aerial vehicles undoubtedly a good option as their 6 DoF enable the exploration system to observe a zone from almost any point of view, ensuring more complete coverage of the scene. In addition, their flying ability and ground independence allow a more efficient path planning. Moreover, the algorithms must run onboard a machine that normally does not have cutting edge technical specifications so the efficiency is of particular importance.

Surmann et al. pioneered the 3D mapping approach. In [137] they present an automatic system for gauging and digitalization of 3D indoor environments, but the navigation of the robot keeps in the 2D ground plane. They perform it splitting the development into three modules. The first one registers the 3D scans and relocalizes the robot using a fast variant of the Iterative Closest Points (ICP) algorithm. In the second module, an NBV planner, computes the next nominal pose based on the acquired 3D data while avoiding obstacles. The third and last module is a closed-loop motor controller that guides the mobile robot to a pose based on odometry while avoiding collisions with dynamical obstacles. The exploration candidates are, initially, randomly generated poses that are reduced based on: the evaluated information gain value, determined by the number of intersections with frontier lines; the distance to the current position; and the angle to the current position. The exploration terminates if the set of candidate viewpoints is empty. Surmann et al. perform the 3D digitalization using a fast octree-based [138] visualization method, but the navigation of the robot is performed in a 2D map. This difference can lead to inconsistencies in some situations. 2D map-based systems of exploration may mark as free space areas where the 3D system can detect an area which the robot cannot navigate through. For example, negative obstacles (defined as obstacles below the floor plane such as down steps, ditches, or cliffs) should not exist in the environment where a system performs an exploration procedure taking into account only 2D information.

A different matter that affects the computational requirements, namely the memory needs, is the inability to run any exploration process offline. The robot actions are calculated on the go, opposite to the legacy SLAM, the robot’s full path is unknown at runtime and so it is the final size of the map. To deal with this limitation, Feder et al. [4] shows an implementation that initializes new features into the map, matches measurements to these features, and deletes out-of-date ones using a delayed nearest neighbor data association strategy. They introduce a method for performing adaptive SLAM in unknown environments for any number of features. It is based on choosing actions that, given the current sensor measurements and map/robot state, would maximize the information gained in the next measurement. The technique is oriented for local adaptive mapping and navigation as, at each cycle, only the next action of the robot is considered. It can be formulated globally by predicting over an expanded time horizon, as the computational cost grows tremendously.

Building autonomously an accurate 3D representation of a whole indoor environment can be unfeasible as the complexity of the problem increases exponentially based on the size of the environment and the resolution of the map. Besides, it can also be unnecessary to map the entire area as the operational space of the robot can be much smaller and clearly limited. So, systems that can map independently specific rooms or areas are of great interest in many contexts. Maurović et al. [89] present a combination of local and global mapping with a single global navigation method. They combine both 2D and 3D exploration in a cyclic method, enabling tracking of three-dimensional information of large environments to find unexplored volumes. The exploration starts using only 2D measurements and then, the robot follows the jump edges until an enclosed space is detected, i.e., a room. At this point, it switches to the 3D exploration, which takes into account the whole 3D environment information captured by a laser point scanner. The 3D exploration algorithm is focused on the detected room as a small unit of the large environment. When it is explored, the 3D exploration process terminates and exploration continues again with the 2D-based strategy, going back to the first phase. For the individual room 3D exploration, the algorithm of Blaer and Allen [135] is used, which requires a 2D map of the environment in advance and then it steers the exploration towards the most unseen area. That area is determined by the number of unseen voxels at the height of the sensor and in its field of view (FOV).

In contrast, to address the computation and memory limitations of the MAVs mentioned above, Shen et al. [139] propose a stochastic differential equation-based exploration algorithm that considers only the known occupied space in the current map, avoiding the explicit representation of free and unknown space. They determine regions for further exploration based on the evolution of a stochastic differential equation that simulates the expansion of a particle system with Newtonian dynamics.

## 7. ASLAM Method Summary

The main features of the methods presented in the previous sections are grouped in Table 1. For each work, the robot used, the sensor from which the information is gathered, how the world is represented, the core concept of the contribution, the optimization objective and where the test is performed are shown. Optimization column means if the method finds the optimal pose, trajectory, or both for exploration. Despite it would be desirable in a survey to show some performance measures, it is not possible at this stage of development. Few approaches that report performance information are evaluated using different metrics (time/iterations, cells/m^3^/neighbors...). Besides, the algorithms’ performance is strongly dependent on the hardware used and the environment being mapped.

## 8. On Going Developments

While many approaches focus on the coverage and the consistency of the obtained map, they do not ensure a certain quality or completeness of the objects in the scene. Some advances in this area have taken place in the context of 3D reconstruction [86,140,141], which may be helpful in ASLAM solutions. For example, Calli et al. [142] propose an active vision strategy based on extremum seeking control (ESC) where a previous model of the surroundings is not required. Although it is focused on viewpoint optimization for object recognition and grasping in unstructured environments, the continuous ESC algorithm addresses the problem of objective value optimization when the objective function, its gradient and the optimum value are unknown [143] and, thus, it can be used in the optimization of the utility function in an ASLAM approach.

Using semantic information in ASLAM is a new research line where many recent works are focusing on [144]. Having semantic attributes distinction among objects instead of only geometric entities is necessary so the robot can understand the scene surrounding it. This capability has brought big improvements in classical SLAM [145,146,147] and can be particularly useful to explore unknown regions. Ekvall et al. [148] integrate an object recognition system into SLAM in a service robot scenario. The map is built automatically during navigation and it is augmented by adding the objects detected in this process to it. Similarly, Wu et al. [149] fuse semantic information into an object SLAM system, but they actively optimize the motion of the robot to reduce the observation uncertainty on target objects and increase their pose estimation accuracy. They propose an object-driven exploration strategy that takes into account the completeness of object observation and pose estimation uncertainty, which significantly improves the accuracy of the generated object map.

Deep reinforcement learning is another research line that should be explored here, as it is having promising results in many fields and the ASLAM model fits with the problem type to which it gives response [150,151,152,153,154,155]. Although not many machine learning approaches have been published yet in the ASLAM field, the proposals of recent years have drawn the attention of the community [156,157,158,159,160]. As mentioned in Section 2, surveillance and exploration approaches differ in the key point that in the former problem the environment is known since the beginning and later, it must be discovered. Nevertheless, they have some similarities and a single improvement can affect both research lines. For example, Ly and Tsai [161] propose a greedy and supervised learning approach for visibility-based exploration, reconstruction and surveillance. Given the set of previously-visited points, they compute the cumulative visibility and frontiers. They train a convolutional neural network that learns geometric priors for a large class of obstacles, increasing the efficiency at runtime. Then, they approximate the gain function by applying the trained neural network on this pair of inputs, and pick the next point according to [83]. This procedure is repeated until there are no frontiers or occlusions. Although it is mainly focused in surveillance, it also addresses exploration by learning the parameters of a function using only the observations as input.

### Open Research Questions

ASLAM is still far from being a solved problem that can be used effectively in nearly any environment. Although there are plenty of products that perform SLAM both in domestic and industrial scenarios, none of them offers the functionality of creating the map autonomously without human intervention. There are yet some questions that must be tackled.

In any state, the robot might have the possibility to perform multiple actions depending on the inputs received. Thus, the ability to foresee the effect of each individual action is a key point in the decision-making process [162]. Besides, each action should contribute to the mapping procedure and it may alter the contribution that the consequent actions make. Optimizing this function in the process of mapping an unknown environment, where the objective model and the time needed to build it are unknown, is still under research. Though the process of predicting the future impact of an action is computationally expensive, there are recent advancements by using spectral techniques [163] and deep learning [164].

When to finish the mapping process is another essential aspect that needs to be answered yet. Many of the termination criteria used, such as the number of scans, the time elapsed, distance traveled, and more are hardly dependent on the environment is being modeled and thus are usually set based on trial and error. At some point in the mapping, too much information will only lead to contradictory results and might end up in non-recoverable states due to several wrong loop closures. If the method focuses on coverage, it may be easier to decide correctly when the process can be considered as finished, but this gets more complex as the required quality of the resulting representation increases. The balance between exploration and exploitation has a huge impact throughout the whole process.

Generally, the map created in a classical SLAM procedure needs a post-processing step in which erroneously added elements are deleted. Normally, this addition is not an error of the algorithm itself. The elements removed are usually objects that, without being dynamic, have their position and orientation modified in everyday use (e.g., a chair). That is, these refining actions are based on the human experience and robots have not that knowledge yet. Giving an ASLAM system the ability to take these decisions while creating the map and avoiding the necessity of post-processing is required when the robot starts navigation tasks automatically once the mapping step is considered as completed. Semantic information processing is showing some promising results in this field [165].

## 9. Conclusions

This survey reviews the major ASLAM techniques in the field of mobile robotics. Most of the studied approaches assume the system operates in an indoor static environment, being ground and aerial robots the main actors in 2D and 3D ASLAM systems, respectively. There are some research works trying to solve the problem in other scenarios as well, such as in underwater environments [123,166,167,168].

For 2D map-based techniques, frontier-based approaches are the most used ones. In the standard algorithm, the robot just navigates to the optimal frontier point. A mobile robot acquires information and increases localization drift while moving. Many approaches add constraints to guarantee that, for example, the uncertainty in the localization is low or the system always navigates to the optimal frontier viewpoint. However, these algorithms keep being very dependent on the sensor and map representation used. With 3D data, by contrast, receding horizon-based systems are having a greater impact. They take into account the information gain along the whole path, and are easy to adapt to any sensor configuration. Besides, the computational complexity is low comparing with the expensive frontier clustering, which eases applications in environments with bigger dimensions. As a counterpart, RH-NBV approaches may get stuck in local minima. With respect to world representations, occupancy grid maps predominate, specially octree-based structures. Works that include deep learning models and semantic data structures are showing promising results and they may improve considerably the ASLAM procedures but there are not enough publications yet to draw clear conclusions.

A comparison of the advantages and disadvantages among the different approaches remains. In order to perform such a comparison, code availability is mandatory. This will allow to execute different approaches using a robot platform in a concrete environment and to measure features such as efficiency, mapping completion and accuracy.

It can be concluded that the ASLAM topic has evolved so rapidly in very few years, still having much to offer to the field of mobile robot navigation.

## Figures and Tables

**Table 1:** Main features of representative ASLAM methods.

Authors	Robot and Sensor	World Representation	Main Technique	Optimisation	Test
Yamauchi [64]	Ground robot with sonar	Occupancy grid map	Frontier exploration	Pose	Real environment
Feder et al. [4]	Nomad Scout robot with sonar	Metric, feature-based map	Delayed nearest neighbour	Pose	Simulation
González-Baños and Latombe [124]	Nomadic SuperScout robot with LIDAR	Polygonal map	NBV	Pose	Simulation + real environment
Bourgault et al. [60]	Ground robot with LIDAR	Grid map	Shannon information gain	Pose	Real environment
Surmann et al. [137]	Ariadne robot with 3D LIDAR	Metric, feature-based map + 3D octree-based visualisation	NBV	Pose	Real environment
Stachniss et al. [125]	Pioneer II with LIDAR	Occupancy grid map	Active loop-closing	Pose	Real environment
Stachniss et al. [80]	Pioneer II with LIDAR	Occupancy grid map	Rao-Blackwellized particle filter	Pose	Simulation + real environment
Joho et al. [75]	Pioneer II with LIDAR	MLS map	Expected information gain with 3D ray-casting	Pose	Simulation + real environment
Maurović et al. [89]	Ground robot with 3D LIDAR (2D + pan-tilt)	Occupancy grid map + 3D polygonal, feature-based map	NBV with room detection	Pose	Simulation + real environment
Carlone et al. [78]	Pioneer P3-DX with LIDAR	Occupancy grid map	Kullback-Leibler divergence	Pose + trajectory	Simulation
Trivun et al. [82]	Pioneer 3-DX with LIDAR	Occupancy grid map	Frontier exploration with FastSLAM	Pose	Simulation + real environment
Zhu et al. [99]	EPIA-P910 MAV with RGBD 3D sensor	OctoMap	Frontier exploration	Pose	Real environment
Bircher et al. [83]	AscTec Firefly MAV with 3D RGBD/LIDAR	OctoMap	RRT	Trajectory	Simulation + real environment
Senarathne and Wang [101]	Pioneer3-AT and Husky A200 robots with 3D RGBD sensor and 2D LIDAR	OctoMap	Surface frontiers-based NBV	Pose	Simulation + real environment
Papachristos et al. [112]	Hexarotor UAV with stereo camera pair	OctoMap	RRT	Pose + trajectory	Simulation + real environment
Umari and Mukhopadhyay [62]	Kobuki with 2D LIDAR	Occupancy grid map	Multiple RRT	Pose	Simulation + real environment
Valencia and Andrade-Cetto [127]	2D LIDAR	Occupancy grid map	Pose SLAM	Pose + trajectory	Simulation
Bircher et al. [110]	AscTec Firefly hexacopter MAV with a Visual-Inertial Sensor	OctoMap	Receding horizon path planning	Pose + trajectory	Simulation + real environment
Selin et al. [63]	Drone with 3D RGBD	OctoMap	Receding Horizon Next-Best-View planning with Rapidly-exploring Random Trees (RRT) + Frontier exploration	Pose + trajectory	Simulation + real environment
Faria et al. [115]	UAS with two RGBD cameras	OctoMap	Lazy Theta* path planning	Pose + trajectory	Simulation + real environment
Papachristos et al. [113]	AscTec Firefly hexacopter MAV with RGBD sensor	OctoMap	Receding Horizon Next-Best-View Planner, with the extension of Localization Uncertainty-aware Planning	Pose + trajectory	Simulation + real environment
Suresh et al. [123]	Autonomous underwater vehicle (HAUV) with sonar	OctoMap	Receding horizon ”Next-Best-View” planner	Pose	Simulation + real environment
Dai et al. [84]	AscTec Firefly hexacopter MAV with a Visual-Inertial Sensor	OctoMap	Hybrid between frontier-based and sampling-based exploration	Pose	Simulation + real environment

**Figure A1:**
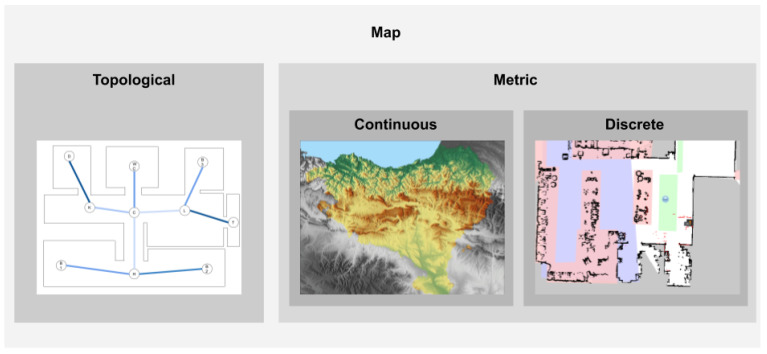
Map representation types.

## Data Availability

Not applicable.

## Abbreviations

The following abbreviations are used in this manuscript:

2D	Two-Dimensional
3D	Three-Dimensional
ASLAM	Autonomous Simultaneous Localization And Mapping
BFS	Breadth-First Search
CML	Concurrent Mapping and Localization
DoF	Degrees of Freedom
DWA	Dynamic Window Approach
EKF	Extended Kalman Filter
ESC	Extremum Seeking Control
FoV	Field of View
GPS	Global Positioning System
ICP	Iterative Closest Points
IMU	Inertial Measurement Unit
LIDAR	Light Detection and Ranging or Laser Imaging Detection and Ranging
LoS	Line of Sight
MAV	Micro Aerial Vehicle
MLS	Multi-Level Surface
NBV	Next Best View
POMDP	Partially Observable Markov Decision Process
RH	Receding Horizon
RH-NBV	Receding Horizon Next Best View
ROS	Robot Operating System
RRT	Rapidly-exploring Random Tree
SfM	Structure from Motion
SLAM	Simultaneous Localization And Mapping
SPLAM	Simultaneous Planning, Localization and Mapping
TOED	Theory of Optimal Experimental Design
UAV	Unmanned Aerial Vehicle

## Appendix A. World Representation

Environment representation in ASLAM does not differ from conventional SLAM systems, as shown in Figure A1. Many other works already explain the different methods to model geometry in a robot readable format [6,15,39,59,169,170] and the IEEE Map Data Representation working group developed a standard specification for representing a map used for robot navigation [171].

There are two predominant map representations: topological maps and metric maps. The first ones, similar to graphs, only consider places and relations between them, represent the environment as a set of nodes (locations) that are connected by edges. An edge between two nodes is labeled with a probability distribution over the relative locations of the two poses, conditioned to their mutual measurements [172]. A representation of the world based on this simplification eases mapping large extensions and provides all the necessary information for path planning [8]. Notwithstanding the simplification of the world model, the concept of vicinity is lost in topological representations not to mention the absence of explicit information about the occupancy of the space. To overcome this, many authors store additional information or use a combination with metric maps [173,174,175,176,177].

Alternatively, in metric maps the objects are placed with precise coordinates. They provide all the necessary information to apply a mapping or navigation algorithm, but the map size is directly proportional to the size of the working area, which makes, by itself, costly to model large areas specially concerning 3D mapping. Three are the common representations used in metric maps: landmark-based maps, occupancy grid maps and geometric maps.

Landmark-based representations, also called feature-based representations, identify and keep the poses of certain distinctive elements [178,179]. A requirement that must be met in these representation is that the landmarks must be unique and distinguishable by the robot perception system. These landmarks can be points, lines or corners, or even faces in 3D mapping, forming a sparse representation of the scene. Landmarks can be more than raw sensor data measurements, such as complex descriptors which establish the corresponding data association with each measurement.

Instead, occupancy grid maps discretize the environment into so-called grid cells. Each cell stores information about the area it covers. It is common to store in each cell a single value representing the probability of being an obstacle there. Grid cells may contain 2D or 3D information [180,181]. There is a variant referred as 2.5D that, without being a pure 3D grid map, stores height information in an extended 2D grid cell map [182]. Grid maps can be formed by regular grids or by sparse grids. Regular grids make a discretization of the continuous space into cells that always have the same dimensions, while sparse grids extend the concept of the regular grid by grouping regions with same values, similar to a tree-like approach. In this occupancy grid classification, a 3D technique that it is worth mentioning is the Octree Encoding [138]. An octree is a hierarchical 8-ary tree structure that can represent objects of any morphology in any specified resolution. As it is shown later, it is widely used in systems that require 3D data storage due to its high efficiency, because the memory required for representation and manipulation is on the order of the surface area of the object. A well known implementation of this idea is the OctoMap framework (https://octomap.github.io/, accessed on 11 February 2021) developed by Kai M. Wurm and Armin Hornung [183].

A widely used concept in grid maps is that of the costmap. The costmap represents the difficulty of traversing different areas of the map. It takes the form of an occupancy grid with abstract values that do not represent any measurement of the world, as the cost itself is obtained by combining the static map, the local obstacle information and the inflation layer. It is usually used for path planning [184,185,186,187].

Finally, there are geometric maps, which can be considered also discrete maps. In geometric maps all the objects detected by the sensors are represented by simplified geometric shapes, such as circles or polygons. This is an attempt to efficiently represent the environment without losing much information, but it hampers trajectory calculation and the overall management of the data. So, in practice, not many works make use of this method and the occupancy grid map option is preferred [188,189].

All the same, additional data for active mapping purposes, e.g., information gain values, may also be stored, which opens up a wide range of possibilities for designing ASLAM algorithms [190]. The ASLAM approaches may vary greatly upon the representation being used, but the most significant difference is if they are 2D or 3D oriented, or a mixture of both.
